# Systematic evaluation of cancer‐specific genetic risk score for 11 types of cancer in The Cancer Genome Atlas and Electronic Medical Records and Genomics cohorts

**DOI:** 10.1002/cam4.2143

**Published:** 2019-04-09

**Authors:** Zhuqing Shi, Hongjie Yu, Yishuo Wu, Xiaoling Lin, Quanwa Bao, Haifei Jia, Chelsea Perschon, David Duggan, Brian T. Helfand, Siqun L. Zheng, Jianfeng Xu

**Affiliations:** ^1^ Program for Personalized Cancer Care NorthShore University HealthSystem Evanston Illinois; ^2^ State Key Laboratory of Genetic Engineering, School of Life Science Fudan University Shanghai China; ^3^ Fudan Institute of Urology, Huashan Hospital, Fudan University Shanghai China; ^4^ Translational Genomics Research Institute Phoenix Arizona

**Keywords:** age at diagnosis, cancer, genetic risk score

## Abstract

**Background:**

Genetic risk score (GRS) is an odds ratio (OR)‐weighted and population‐standardized method for measuring cumulative effect of multiple risk‐associated single nucleotide polymorphisms (SNPs). We hypothesize that GRS is a valid tool for risk assessment of most common cancers.

**Methods:**

Utilizing genotype and phenotype data from The Cancer Genome Atlas (TCGA) and Electronic Medical Records and Genomics (eMERGE), we tested 11 cancer‐specific GRSs (bladder, breast, colorectal, glioma, lung, melanoma, ovarian, pancreatic, prostate, renal, and thyroid cancer) for association with the respective cancer type. Cancer‐specific GRSs were calculated, for the first time in these cohorts, based on previously published risk‐associated SNPs using the Caucasian subjects in these two cohorts.

**Results:**

Mean cancer‐specific GRS in the population controls of eMERGE approximated the expected value of 1.00 (between 0.98 and 1.02) for all 11 types of cancer. Mean cancer‐specific GRS was consistently higher in respective cancer patients than controls for all 11 types of cancer (*P* < 0.05). When subjects were categorized into low‐, average‐, and high‐risk groups based on cancer‐specific GRS (<0.5, 0.5‐1.5, and >1.5, respectively), significant dose‐response associations of higher cancer‐specific GRS with higher OR of respective type of cancer were found for nine types of cancer (*P_‐trend_* < 0.05). More than 64% subjects in the population controls of eMERGE can be classified as high risk for at least one type of these cancers.

**Conclusion:**

Validity of GRS for predicting cancer risk is demonstrated for most types of cancer. If confirmed in larger studies, cancer‐specific GRS may have the potential for developing personalized cancer screening strategy.

## 1 INTRODUCTION

Cancer is a major public health issue in the United States and across the world. Based on the projection of the National Institute of Health, an estimated 1 735 350 new cases of cancer will be diagnosed in the United States and 609 640 people will die from the disease in 2018.^1^ Although most cancer patients do not have germline mutations in known major cancer susceptibility genes, inherited risk factors play an important role in the development of cancer. This notion is supported by many genetic studies, including two large twin studies in Nordic countries.^2^, ^3^ In a prospective study of 80 309 monozygotic and 123 382 same‐sex dizygotic twin individuals within the population‐based registers of Denmark, Finland, Norway, and Sweden,^3^ Muccia and colleagues found that heritability (ie, the proportion of variability in disease risk in a population due to genetic factors) of cancer overall was 33%. Significant heritability was observed for the cancer types of skin melanoma (58%), prostate (57%), nonmelanoma skin (43%), ovary (39%), kidney (38%), breast (31%), and corpus uteri (27%). In addition to germline mutations in known cancer susceptibility genes that account for a small proportion of heritability, it is hypothesized that polygenic inheritance (ie, many common but small‐effect genetic variants) also contributes significantly to heritability.

Genome‐wide association studies (GWAS) in the last decade have successfully identified several hundreds of cancer‐specific risk‐associated SNPs.^4^, ^5^ Although the biological mechanisms for these SNPs are largely unknown at this stage, the associations are most likely valid due to the stringent criteria for declaring statistical significance (*P* < 5 × 10^‐8^) and requirement of validation in independent study populations. Individually, these SNPs have a moderate effect on disease risk; with odds ratios (OR) typically ranging from 1.1‐1.5. However, when more than one risk‐associated SNP is inherited in an individual, they can have a cumulative, clinically significant effect on disease risk.^6^ Polygenic risk scores can now identify a substantially larger fraction of the population at comparable or greater disease risk than is found by rare monogenic mutations.^7^

Several polygenic risk score methods have been employed to measure the cumulative effect of multiple risk‐associated SNPs, including (1) a direct risk allele count, (2) an OR‐weighted risk allele count, and (3) using the latter approach but with population standardization, commonly termed as a genetic risk score (GRS).^8^ The mean of score from the first two methods will vary depending on the number of risk‐associated SNPs used in calculation. In contrast, because GRS is population standardized for each SNP, its expected mean in the general population will always be 1.00 regardless of the number of SNPs used in calculation. Furthermore, GRS values can be simply interpreted as relative risk to the general population. These two important features of GRS make it easy to implement for individual risk assessment.

Published studies to date have consistently demonstrated associations of various polygenic risk scores with risk for several types of cancer.^6^, ^9^, ^10^ However, associations using the population‐standardized GRS have only been reported for a limited number of cancer types such as prostate, breast, and colorectal cancer.^36^, ^37^ We hypothesize that GRS is a valid tool for risk assessment of most common cancers. To test this hypothesis, we systematically assessed associations of 11 cancer‐specific GRSs (bladder, breast, colorectal, glioma, lung, melanoma, ovarian, pancreatic, prostate, renal, and thyroid cancer) with their respective cancer risk. This analysis was performed in two large publicly available cohorts: The Cancer Genome Atlas (TCGA) with various types of cancer patients and the Electronic Medical Records and Genomics (eMERGE) Network with a large number of population controls. Results from this study may provide important information for GRS to be used for inherited risk assessment.

## 2 METHODS

### 2.1 Study subjects and genotyping data

We requested access of these two study cohorts through dbGaP. TCGA is a comprehensive and coordinated effort by the National Institutes of Health (NIH) to accelerate understanding of the molecular basis of cancer through the application of genome analysis technologies, including SNP genotyping. TCGA includes more than 11 000 patients of 33 types of cancer. In this study, we analyzed 11 types of solid tumor cancer where at least six cancer‐specific risk‐associated SNPs were available. We limited the association analysis in Caucasians due to most study subjects (85%) being of Caucasian decent. Genotyping data from the Affymetrix Genome‐Wide Human SNP Array 6.0 are available.

Electronic Medical Records and Genomics is a consortium of five participating sites (Group Health Seattle, Marshfield Clinic, Mayo Clinic, Northwestern University, and Vanderbilt University) funded by the National Health Genome Research Institute (NHGRI) to investigate the use of electronic medical record systems for genomic research.^43^ The goal of eMERGE is to conduct GWAS in approximately 19 000 individuals using electronic medical record (EMR)‐derived phenotypes and DNA from linked biorepositories. Genotyping data from the Illumina Human660W‐Quad v1.0 BeadChip are available. Because subjects in eMERGE were not recruited for specific for cancer studies, we treated them as population controls. We did not include a subset of cohort (N = 1700) that was only approved for dementia study. To match race of subjects in TCGA, only Caucasian subjects were included in the analysis (79% of eMERGE subjects were Caucasians).

### 2.2 Ancestry analysis and SNP imputation

We inferred ancestry information of study subjects in TCGA and eMERGE based on available genotyping data in the SNP arrays using the ADMIXTURE computer program.^44^ Subjects with the estimated proportion of Caucasian ancestry >60% were considered as Caucasians. We also estimated the eigens of these subjects using the EIGENSOFT (Version 3.0) and plotted the first two eignes of these subjects as well as Caucasians, African Americans, and East Asians subjects from the 1000 Genome Project.^45^, ^46^ All Caucasian subjects in the TCGA cohort fell in the cluster of Caucasians (Figure S1).

For risk‐associated SNPs that were not included in the downloaded data file, presumably because they were not found on the original genotyping array, imputation was performed using IMPUTE 2.2.2 based on the combined data of the 1000 Genomes Project and HapMap3 data.^47^ A posterior probability of >0.9 was applied to all imputed genotypes.

### 2.3 Risk‐associated SNPs

Cancer‐specific risk‐associated SNPs were cataloged based on GWAS papers of the 11 types of cancer published prior to July 1, 2018. The following criteria were used to select independent and reliable risk‐associated SNPs: (1) discovered from GWAS studies of Caucasian subjects, with at least 1000 cases and 1000 controls in the first stage; (2) confirmed in additional stages with combined *P* < 5 × 10^‐8^; and (3) independent, linkage disequilibrium (LD) measurement (*r* ^2^ <0.2) between any pair of SNPs. Risk‐associated SNPs available directly and indirectly (from imputation) in the TCGA and eMERGE are presented in Table S1, including 10, 66, 30, 19, 6, 17, 11, 9, 79, 10, and 6 SNPs for bladder,^48^, ^49^ breast,^52^, ^53^ colorectal,^21^, ^59^, ^60^ glioma,^70^, ^71^ lung,^73^, ^74^ melanoma,^78^, ^79^ ovarian,^84^, ^85^ pancreatic,^89^, ^90^ prostate,^31^, ^32^, ^33^, ^92^ renal,^97^, ^98^ and thyroid cancer,^102^, ^103^ respectively.

### 2.4 GRS calculation

GRS, an OR‐weighted and population‐standardized polygenic risk score, was computed using allelic ORs obtained from the external studies and allele frequencies in the gnomAD (NFE population).^8^ Briefly, GRS was calculated by multiplying the per‐allele OR for each SNP and normalized by the expected risk effect of each SNP in the population (*W*).

$$\text{GRS}=\prod_{i=1}^{n}\frac{OR_{i}^{g_{i}}}{W_{i}}$$

$$W_{i}=f_{i}^{2}OR_{i}^{2}+2f_{i}\left(1-f_{i}\right)OR_{i}+{\left(1-f_{i}\right)}^{2}$$

where, *g*_i _stands for the genotype of SNP *i* in an individual (0, 1, or 2 risk alleles), *OR_i_* stands for the allelic OR of SNP *i*, and *f_i_* stands for the risk allele frequency of SNP *i*. Based on the GRS formula, the mean GRS should be 1.00 in the general population and GRS can be interpreted as relative risk to the general population regardless of the number of SNPs used in the calculation.

### 2.5 Statistical analysis

The Wilcoxon rank sum test was used to compare mean cancer‐specific GRS in respective cancer patients and controls. Subjects were categorized into low‐, average‐, and high‐risk groups based on their respective cancer‐specific GRS (<0.5, 0.5‐1.5, and >1.5, respectively). The trend of increasing OR for cancer among subjects in low‐, average‐, and high‐risk groups was tested using a proportion trend test. All statistical tests were performed using R package (Version 3.5.2).

## 3 RESULTS

A total of 5871 Caucasian patients diagnosed with one of the 11 types of cancer in the TCGA and 13 427 Caucasian controls from eMERGE were included in this analysis. The key demographic and clinical information for these study subjects are presented in Table 1. For breast and ovarian cancer, only female patients were included and for prostate cancer, only male patients were included.

**Table 1:** Key demographic and clinical information of study subjects

Cancer type/control group	Sample size (N)	Age at diagnosis (Mean ± SD)	Male (%)
Bladder	343	69 ± 10	74.34%
Breast	827	60 ± 13	0.00%
Colorectal	387	68 ± 13	52.97%
Glioma	992	52 ± 16	58.76%
Lung	908	67 ± 9	60.90%
Melanoma	450	59 ± 16	61.78%
Ovarian	531	60 ± 12	0.00%
Pancreatic	163	66 ± 11	55.21%
Prostate	421	62 ± 7	100.00%
Renal	453	62 ± 12	67.11%
Thyroid	387	49 ± 16	27.39%
eMERGE	13 427	–	47.72%

The mean cancer‐specific GRSs approximated the expected value of 1.00 in the population controls of eMERGE for all 11 types of cancer (Table 2); the mean GRSs ranged from 0.98 (glioma bladder, and thyroid cancer) to 1.02 (melanoma, ovarian, and pancreatic cancer). Mean cancer‐specific GRS values were significantly higher among respective cancer patients in TCGA than controls in eMERGE for all 11 types of cancer (*P* < 0.05) (Table 2).

**Table 2:** Cancer‐specific genetic risk score in cases and controls

Cancer type	SNPs (N)	Mean of GRS (95% CI)		*P*
		Cases	Controls	
Bladder	10	1.04 (1‐1.08)	0.98 (0.97‐0.98)	3.77E‐03
Breast	66	1.15 (1.11‐1.2)	1.01 (1‐1.03)	1.48E‐14
Colorectal	30	1.08 (1.04‐1.12)	1 (0.99‐1.01)	8.29E‐06
Glioma	19	1.22 (1.18‐1.26)	0.98 (0.97‐0.99)	1.39E‐37
Lung	6	1.01 (0.99‐1.02)	0.99 (0.98‐0.99)	1.16E‐02
Melanoma	17	1.2 (1.14‐1.26)	1.02 (1.01‐1.03)	5.99E‐11
Ovarian	11	1.12 (1.08‐1.16)	1.02 (1.01‐1.03)	1.45E‐04
Pancreatic	9	1.13 (1.07‐1.18)	1.02 (1.02‐1.03)	1.45E‐04
Prostate	79	1.3 (1.21‐1.38)	0.99 (0.98‐1.01)	2.07E‐18
Renal	10	1.09 (1.06‐1.12)	1.01 (1‐1.01)	8.66E‐10
Thyroid	6	1.09 (1.04‐1.15)	0.98 (0.98‐0.99)	3.64E‐05

CI, confidence interval; GRS, genetic risk score.

Subjects were then categorized into low‐, average‐, and high‐risk groups for each type of cancer based on their respective cancer‐specific GRS (<0.5, 0.5‐1.5, and >1.5, respectively). Compared to subjects with average‐risk, subjects classified as high‐risk had OR >1 for their respective type of cancer in 10 types of cancer; nine of which reached statistically significant level (*P* < 0.05) (Table 3). Conversely, compared to subjects with average‐risk, subjects classified as low‐risk had OR <1 for their respective type of cancer in 10 types of cancer; seven of which reached statistically significant level (*P* < 0.05). A significant dose‐response association of higher cancer‐specific GRS with higher odds ratio of respective type of cancer was found for nine types of cancer (*P_‐trend_* < 0.05).

**Table 3:** Odds ratio for each type of cancer among subjects classified as low‐ and high‐risk based on cancer‐specific genetic risk score

Cancer type	Low‐risk			Average‐risk		High‐risk			
	Sample size (case/control)	OR (95% CI)	*P*	Sample size (case/control)	OR	Sample size (case/control)	OR (95% CI)	*P*	*P‐trend*
Bladder	7/279	1.02 (0.48‐2.18)	0.96	301/12245	1.00	35/903	1.58 (1.1‐2.25)	0.01	0.02
Breast	68/1064	0.54 (0.42‐0.71)	3.42E‐06	572/4874	1.00	187/1082	1.47 (1.23‐1.76)	1.80E‐05	5.02E‐15
Colorectal	15/687	0.76 (0.45‐1.29)	0.31	324/11324	1.00	48/1416	1.18 (0.87‐1.61)	0.28	0.11
Glioma	75/2198	0.48 (0.37‐0.61)	9.49E‐10	667/9298	1.00	250/1931	1.8 (1.55‐2.1)	2.24E‐14	4.49E‐31
Lung	0/14	0 (0‐NaN)	0.33	886/13044	1.00	22/369	0.88 (0.57‐1.36)	0.56	0.68
Melanoma	22/1227	0.57 (0.37‐0.88)	0.01	323/10262	1.00	105/1938	1.72 (1.37‐2.16)	1.80E‐06	4.13E‐09
Ovarian	10/320	0.43 (0.23‐0.82)	0.01	422/5858	1.00	99/842	1.63 (1.3‐2.06)	2.64E‐05	9.90E‐08
Pancreatic	0/399	0 (0‐NaN)	0.03	136/11642	1.00	27/1386	1.67 (1.1‐2.53)	0.02	9.20E‐04
Prostate	36/1274	0.43 (0.3‐0.62)	1.76E‐06	268/4098	1.00	117/1035	1.73 (1.38‐2.17)	1.93E‐06	4.02E‐16
Renal	1/200	0.15 (0.02‐1.08)	0.03	409/12401	1.00	43/826	1.58 (1.14‐2.18)	0.01	4.29E‐04
Thyroid	21/1063	0.72 (0.46‐1.12)	0.15	303/11020	1.00	63/1344	1.7 (1.29‐2.25)	1.37E‐04	3.55E‐05

CI, confidence interval; OR, odds ratio.

We further estimated the proportion of high‐risk subjects in the population controls of the eMERGE cohort. At the individual cancer type level, the proportion of subjects that were classified into high‐risk ranged from 2.75% (lung cancer) to 16.15% (prostate cancer) (Table 4). When all 11 types of cancer were tallied together, 64% (61% in male, 66% in female) of subjects were classified as high‐risk for at least one type of cancer. 49.50% (49.52% in male, 49.47% in female) of subjects were classified as low‐risk for at least one type of cancer, and 84.55% (83.85% in male, 85.19% in female) of subjects were classified as either high‐risk or low‐risk for at least one type of cancer.

**Table 4:** Proportion of subjects in each risk category in eMERGE

Cancer type	Sample size (N)	Low‐risk (GRS <0.5)	Average‐risk (GRS:0.5‐1.5)	High‐risk (GRS >1.5)
Bladder	13 427	2.08%	91.20%	6.73%
Breast	7020	15.16%	69.43%	15.41%
Colorectal	13 427	5.12%	84.34%	10.55%
Glioma	13 427	16.37%	69.25%	14.38%
Lung	13 427	0.10%	97.15%	2.75%
Melanoma	13 427	9.14%	76.43%	14.43%
Ovarian	7020	4.56%	83.45%	11.99%
Pancreatic	13 427	2.97%	86.71%	10.32%
Prostate	6407	19.88%	63.96%	16.15%
Renal	13 427	1.49%	92.36%	6.15%
Thyroid	13 427	7.92%	82.07%	10.01%

GRS, genetic risk score.

## 4 DISCUSSION

This is the first systematic evaluation of cancer‐specific and population‐standardized GRS for risk assessment of multiple types of cancer and the first study to examine this risk in publicly available study cohorts (TCGA and eMERGE). In a recently published seminal study, Fritche and colleagues studied multiple types of cancer in a large phenome‐wide association study (PheWAS) and demonstrated that the top quartiles of cancer‐specific polygenic risk score were significantly higher than the bottom quartile for six types of cancer (breast, prostate, melanoma, basal cell carcinoma, squamous cell carcinoma, and thyroid cancer), with OR >2.^9^ There are many similarities in method, approach, and results between the study described here and their study. Both studies used polygenic risk score methods, adopted multicancer approach, and found evidence that cancer‐specific polygenic risk scores are strongly associated with respective cancer risk for multiple types of cancer. However, there is also a major difference in how the two studies actually calculated the polygenic risk score, which can have major implications in interpretation and translation.

Our method uses a population‐standardized GRS approach. While this difference—population‐standardized versus not—does not affect the performance comparison between cases and controls in a study cohort because the score ranking order of subjects is the same in both methods,^8^ the score values of nonpopulation‐standardized methods—for example, top 25%—are not practically meaningful for individuals seen in a clinic. In contrast, because GRS is relative risk to the general population, its values are meaningful for individual subjects and can be used directly to stratify individuals’ risk. There are two additional advantages for population‐standardized GRS. First, with the expected mean GRS value of 1.00 in the general population, it provides an objective tool to assess the performance of GRS. Deviation from this property signifies a poor performance of GRS. Second, with GRS, the values represent risk compared to the general population, making it straightforward to identify high‐risk subjects based on subjects’ GRS values.

In this study, we found that the mean cancer‐specific GRSs were significantly higher in respective cancer patients than controls for all 11 evaluated types of cancer. When subjects were categorized into low‐, average‐, and high‐risk groups based on their cancer‐specific GRSs (<0.5, 0.5‐1.5, and >1.5, respectively), a significant dose‐response association of higher cancer‐specific GRS with higher odds ratio of the respective type of cancer was found for eight types of cancer. Furthermore, we found that the mean GRS values approximated their expected value (1.00) in the population controls of eMERGE for all 11 types of cancer. A significant proportion of subjects (64%) can be classified as high risk (GRS >1.5) for at least one type of cancer in the population controls.

The statistical association of GRS with cancer risk from study populations provides broad‐sense validity for its risk stratification. Broad‐sense validity is necessary but insufficient to warrant GRS as a testing tool for individual risk assessment. For individual risk assessment, the validity of specific GRS values (we refer to as narrow‐sense validity) must be met for several reasons. First, in individual testing, only GRS values of test subjects are available, not the percentiles of GRS that are determined based on all subjects in a study cohort. Clinicians treat patients not cohorts. Second, GRS values, not percentiles, are used directly to estimate an individuals’ relative and absolute disease risk including lifetime risk. For example, if a test result provided a prostate cancer GRS value of 1.8 for a 61‐year‐old Caucasian man, we would report that the subject has a 1.8‐fold increased risk for prostate cancer compared to the general population and a 29.6% remaining lifetime risk by age 85 years based on his GRS values, current age, and age‐specific incidence and mortality data of Non‐Hispanic Whites from SEER data (2011‐2015).^106^, ^107^ Therefore, additional evidence related to the narrow‐sense validity is needed before GRS can be used in individual risk assessment.

There are important clinical utilities for risk assessment using GRS. For cancer types where a population screening is recommended, such as prostate, breast, colorectal, and lung cancer, primary care physicians can incorporate GRS to develop a personal screening strategy for the need, timing, and frequency of cancer screenings. This personalized approach is likely to maximize the potential benefits and minimize the potential harms of cancer screening.^109^, ^110^ For example, studies from Frampton et al, showed that personalized screening strategy based on polygenic risk score have the potential to greatly reduce the number of individuals screened while still detecting nearly as many cases.^37^, ^38^ For other types of cancer, medical geneticists and specialists can use GRS to supplement other known risk factors, such as family history and high‐penetrance genes, to better determine the risk for diagnostic workup.

GRS can be used to supplement family history for a better and more comprehensive assessment of an individuals’ risk. These two risk factors have been previously shown to be independent measures of inherited risk. For example, in prostate cancer, family history and a high GRS (>1.4) can identify 17% and 24% of men with high risk for prostate cancer, respectively, in the Prostate Cancer Prevention Trial.^40^ The combination of family history and/or GRS can identify 36% of men at high risk for prostate cancer. The observed prostate cancer risk was 29%, 33%, and 31% for family history alone, GRS alone, and combination of family history and GRS, respectively. GRS has an advantage over family history in that it is an objective measurement of disease risk not susceptible to various issues related to the collection of family history and recall bias. Furthermore, accurate collection of family history is challenging. For example, family history information of specific cancer was not available in these two important study cohorts (TCGA and eMERGE).

The precise reason for weaker associations of GRS with some types of cancer is unknown but may be due to a number of factors, including fewer numbers of risk‐associated SNPs available in this study, and existence of different subtypes of cancer where risk‐associated SNPs and etiology could be different. For example, in the lung cancer cohort, 6, 9, and 15 SNPs were reported to be associated with squamous cell, adenocarcinoma, and overall lung cancer, respectively, and some of these SNPs are overlapped. In this study, we calculated lung cancer GRS using risk‐associated SNPs reported in any type of lung cancer. This approach was taken because of the limited number of patients available for each subtype of cancer (456 squamous cell lung cancer patients and 452 adenocarcinoma lung cancer patients) and only six risk‐associated SNPs in any type of lung cancer were available in both SNP arrays in the TCGA and eMERGE.

A number of additional limitations are noticed in this study. First, the study was limited to Caucasians only, due to the fact that vast majority of study subjects in the TCGA (85%) and eMERGE (79%) are of Caucasian decent. A similar type of analysis should be performed for other racial groups. Second, the sample sizes of patients in TCGA are relatively small, especially for bladder, colorectal, pancreatic, and thyroid cancer (<400). The smaller sample size reduced statistical power in this study. Larger population cohorts and biorepositories, with known case‐control status of multiple cancer phenotypes in various racial groups, are needed to replicate and substantiate our findings. For example, data from the PheWAS of Michigan Genomics Initiative can be used to assess GRS performance of multiple types of cancer.^9^ Third, only a subset of established risk‐associated SNPs were available in this analysis because genotype data was extracted from two earlier versions of SNP arrays (Affymetrix Genome‐Wide Human SNP Array 6.0 and Illumina Human660W‐Quad v1.0 BeadChip). This limitation further reduced the statistical power of our study. Today, low‐coverage (~2x) whole‐genome sequencing (WGS) is a cost‐effective option for obtaining all common variants in the genome, including risk‐associated SNPs to be identified in the future.^113^

In summary, this study provides additional evidence supporting the use of polygenic risk scores for risk stratification and, specifically, the validity of GRS in predicting cancer risk for several types of cancer. If confirmed in larger studies, cancer‐specific GRS may be used for individual risk assessment to develop personalized cancer screening strategy.

## CONFLICT OF INTEREST

NorthShore University HealthSystem has an ongoing research agreement with Ambry Genetics to develop GRS for various common diseases.
