# Supplementary material for: Systematic evaluation of cancer‐specific genetic risk score for 11 types of cancer in The Cancer Genome Atlas and Electronic Medical Records and Genomics cohorts
Source: Cancer Med. 2019 Apr 9;8(6):3196–205. doi: 10.1002/cam4.2143 (PMC6558466; doi:10.1002/cam4.2143)
Supplement: Supplementary file 1 [file CAM4-8-3196-s001.docx]

**Supplementary material**

Figure 1. Eigen plot of subjects from TCGA and the 1000 Genome Project.

Table 1. Characteristics of the selected SNPs for each type of cancer

| Cancer type | SNP | CHR | POS | Gene | Region | Type | RA | RAF^1^ | | | OR^2^ | Reference |
| --- | --- | --- | --- | --- | --- | --- | --- | --- | --- | --- | --- | --- |
|  |  |  |  |  |  |  |  | Case | Control | genomAD |  |  |
| Bladder | rs710521 | 3 | 189645933 | TP63,P3H2 | intergenic | Genotype | T | 0.77 | 0.73 | 0.75 | 1.14 | Figueroa JD.Hum Mol Genet.2014(1) |
| Bladder | rs798766 | 4 | 1734239 | TACC3 | intronic | Genotype | T | 0.21 | 0.20 | 0.20 | 1.22 | Figueroa JD.Hum Mol Genet.2014 |
| Bladder | rs401681 | 5 | 1322087 | CLPTM1L | intronic | Genotype | C | 0.57 | 0.56 | 0.57 | 1.12 | Figueroa JD.Hum Mol Genet.2014 |
| Bladder | rs1495741 | 8 | 18272881 | NAT2,PSD3 | intergenic | Genotype | A | 0.80 | 0.77 | 0.78 | 1.14 | Figueroa JD.Hum Mol Genet.2014 |
| Bladder | rs9642880 | 8 | 128718068 | CASC8,CASC11 | intergenic | Genotype | T | 0.46 | 0.46 | 0.46 | 1.24 | Figueroa JD.Hum Mol Genet.2014 |
| Bladder | rs2294008 | 8 | 143761931 | PSCA | UTR5 | Genotype | T | 0.47 | 0.45 | 0.48 | 1.15 | Wu X.Nat Genet.2009(2) |
| Bladder | rs17674580 | 18 | 43309911 | SLC14A1 | intronic | Impute | T | 0.40 | 0.36 | 0.36 | 1.17 | Rafnar T.Hum Mol Genet.2011(3) |
| Bladder | rs8102137 | 19 | 30296853 | C19orf12,CCNE1 | intergenic | Genotype | C | 0.36 | 0.33 | 0.34 | 1.13 | Figueroa JD.Hum Mol Genet.2014 |
| Bladder | rs62185668 | 20 | 10961935 | LOC101929413,LOC339593 | intergenic | Impute | A | 0.23 | 0.25 | 0.25 | 1.19 | Rafnar T.Hum Mol Genet.2014(4) |
| Bladder | rs1014971 | 22 | 39332623 | CBX6,APOBEC3A_B | intergenic | Genotype | T | 0.66 | 0.64 | 0.64 | 1.13 | Figueroa JD.Hum Mol Genet.2014 |
| Breast | rs10864459 | 1 | 10563609 | PEX14 | intronic | Impute | G | 0.69 | 0.68 | 0.70 | 1.11 | Couch FJ.Nat Commun.2016(5) |
| Breast | rs11552449 | 1 | 114448389 | DCLRE1B | exonic | Impute | T | 0.16 | 0.16 | 0.17 | 1.07 | Michailidou K.Nat Genet.2015(6) |
| Breast | rs12405132 | 1 | 145644984 | NBPF10,NBPF20,RNF115 | intronic | Impute | C | 0.62 | 0.63 | 0.61 | 1.05 | Michailidou K.Nat Genet.2015 |
| Breast | rs6678914 | 1 | 202187176 | LGR6 | intronic | Genotype | G | 0.61 | 0.59 | 0.59 | 1.09 | Michailidou K.Nat Genet.2015 |
| Breast | rs4245739 | 1 | 204518842 | MDM4 | UTR3 | Impute | C | 0.27 | 0.27 | 0.25 | 1.15 | Michailidou K.Nat Genet.2015 |
| Breast | rs12710696 | 2 | 19320803 | NT5C1B-RDH14,MIR4757 | intergenic | Impute | T | 0.39 | 0.36 | 0.36 | 1.10 | Michailidou K.Nat Genet.2015 |
| Breast | rs4577244 | 2 | 29120733 | WDR43 | intronic | Genotype | C | 0.76 | 0.78 | 0.78 | 1.09 | Couch FJ.Nat Commun.2016 |
| Breast | rs4849887 | 2 | 121245122 | LINC01101,GLI2 | intergenic | Genotype | C | 0.90 | 0.89 | 0.91 | 1.10 | Michailidou K.Nat Genet.2015 |
| Breast | rs1550623 | 2 | 174212894 | MLK7-AS1,CDCA7 | intergenic | Genotype | A | 0.85 | 0.84 | 0.85 | 1.06 | Michailidou K.Nat Genet.2013 |
| Breast | rs1830298 | 2 | 202181247 | ALS2CR12 | intronic | Impute | C | 0.30 | 0.28 | 0.27 | 1.06 | Lin WY.Hum Mol Genet.2015(7) |
| Breast | rs13387042 | 2 | 217905832 | LOC101928327,DIRC3-AS1 | intergenic | Genotype | A | 0.55 | 0.52 | 0.52 | 1.14 | Michailidou K.Nat Genet.2015 |
| Breast | rs16857609 | 2 | 218296508 | DIRC3 | ncRNA_intronic | Impute | T | 0.28 | 0.26 | 0.25 | 1.08 | Michailidou K.Nat Genet.2015 |
| Breast | rs6762644 | 3 | 4742276 | ITPR1 | intronic | Genotype | G | 0.40 | 0.38 | 0.38 | 1.07 | Michailidou K.Nat Genet.2015 |
| Breast | rs4973768 | 3 | 27416013 | SLC4A7 | UTR3 | Genotype | T | 0.48 | 0.47 | 0.47 | 1.10 | Michailidou K.Nat Genet.2015 |
| Breast | rs6796502 | 3 | 46866866 | PRSS45,PRSS42 | intergenic | Impute | G | 0.92 | 0.90 | 0.90 | 1.09 | Michailidou K.Nat Genet.2015 |
| Breast | rs9790517 | 4 | 106084778 | TET2 | intronic | Impute | T | 0.26 | 0.22 | 0.24 | 1.05 | Michailidou K.Nat Genet.2015 |
| Breast | rs6828523 | 4 | 175846426 | ADAM29 | intronic | Impute | C | 0.88 | 0.88 | 0.88 | 1.11 | Michailidou K.Nat Genet.2015 |
| Breast | rs2012709 | 5 | 32567732 | ZFR,SUB1 | intergenic | Genotype | T | 0.50 | 0.47 | 0.49 | 1.05 | Michailidou K.Nat Genet.2015 |
| Breast | rs889312 | 5 | 56031884 | C5orf67,MAP3K1 | intergenic | Genotype | C | 0.31 | 0.28 | 0.29 | 1.12 | Michailidou K.Nat Genet.2015 |
| Breast | rs1353747 | 5 | 58337481 | PDE4D | intronic | Genotype | T | 0.91 | 0.90 | 0.90 | 1.09 | Michailidou K.Nat Genet.2015 |
| Breast | rs1432679 | 5 | 158244083 | EBF1 | intronic | Genotype | C | 0.43 | 0.43 | 0.42 | 1.07 | Michailidou K.Nat Genet.2015 |
| Breast | rs11242675 | 6 | 1318878 | FOXQ1,FOXF2 | intergenic | Genotype | T | 0.64 | 0.63 | 0.62 | 1.06 | Michailidou K.Nat Genet.2013(8) |
| Breast | rs204247 | 6 | 13722523 | RANBP9,MCUR1 | intergenic | Genotype | G | 0.43 | 0.43 | 0.44 | 1.05 | Michailidou K.Nat Genet.2015 |
| Breast | rs17529111 | 6 | 82128386 | BCKDHB,FAM46A | intergenic | Impute | C | 0.21 | 0.22 | 0.22 | 1.06 | Michailidou K.Nat Genet.2015 |
| Breast | rs2180341 | 6 | 127600630 | RNF146 | intronic | Impute | G | 0.25 | 0.25 | 0.25 | 1.41 | Gold B.Proc Natl Acad Sci USA.2008(9) |
| Breast | rs12662670 | 6 | 151918856 | CCDC170 | intronic | Impute | G | 0.09 | 0.07 | 0.07 | 1.17 | Michailidou K.Nat Genet.2015 |
| Breast | rs2046210 | 6 | 151948366 | CCDC170,ESR1 | intergenic | Impute | A | 0.35 | 0.35 | 0.32 | 1.08 | Michailidou K.Nat Genet.2015 |
| Breast | rs720475 | 7 | 144074929 | ARHGEF5 | intronic | Genotype | G | 0.77 | 0.74 | 0.75 | 1.06 | Michailidou K.Nat Genet.2015 |
| Breast | rs9693444 | 8 | 29509616 | DUSP4,LINC00589 | intergenic | Genotype | A | 0.33 | 0.32 | 0.32 | 1.07 | Michailidou K.Nat Genet.2015 |
| Breast | rs2943559 | 8 | 76417937 | CASC9,HNF4G | intergenic | Impute | G | 0.08 | 0.08 | 0.07 | 1.13 | Michailidou K.Nat Genet.2015 |
| Breast | rs13267382 | 8 | 117209548 | LINC00536 | ncRNA_intronic | Impute | A | 0.34 | 0.33 | 0.37 | 1.05 | Michailidou K.Nat Genet.2015 |
| Breast | rs11780156 | 8 | 129194641 | MIR1208,LINC00824 | intergenic | Genotype | T | 0.20 | 0.17 | 0.17 | 1.07 | Michailidou K.Nat Genet.2015 |
| Breast | rs1011970 | 9 | 22062134 | CDKN2B-AS1 | ncRNA_intronic | Genotype | T | 0.16 | 0.17 | 0.17 | 1.06 | Michailidou K.Nat Genet.2015 |
| Breast | rs865686 | 9 | 110888478 | KLF4,ACTL7B | intergenic | Genotype | T | 0.65 | 0.63 | 0.64 | 1.11 | Michailidou K.Nat Genet.2015 |
| Breast | rs7072776 | 10 | 22032942 | MLLT10 | downstream | Genotype | A | 0.30 | 0.29 | 0.28 | 1.07 | Michailidou K.Nat Genet.2015 |
| Breast | rs11814448 | 10 | 22315843 | DNAJC1,EBLN1 | intergenic | Impute | C | 0.02 | 0.02 | 0.02 | 1.27 | Michailidou K.Nat Genet.2015 |
| Breast | rs10995190 | 10 | 64278682 | LOC283045,ZNF365 | intergenic | Genotype | G | 0.86 | 0.85 | 0.83 | 1.16 | Michailidou K.Nat Genet.2015 |
| Breast | rs704010 | 10 | 80841148 | ZMIZ1 | intronic | Genotype | T | 0.38 | 0.38 | 0.37 | 1.08 | Michailidou K.Nat Genet.2015 |
| Breast | rs6585202 | 10 | 114782803 | TCF7L2 | intronic | Impute | C | 0.48 | 0.46 | 0.47 | 1.10 | Couch FJ.Nat Commun.2016 |
| Breast | rs11199914 | 10 | 123093901 | MIR5694,FGFR2 | intergenic | Genotype | C | 0.69 | 0.67 | 0.68 | 1.05 | Michailidou K.Nat Genet.2015 |
| Breast | rs1219648 | 10 | 123346190 | FGFR2 | intronic | Genotype | G | 0.43 | 0.39 | 0.39 | 1.31 | Fletcher O.J Natl Cancer Inst.2011(10) |
| Breast | rs3817198 | 11 | 1909006 | LSP1 | intronic | Genotype | C | 0.34 | 0.32 | 0.30 | 1.07 | Michailidou K.Nat Genet.2015 |
| Breast | rs3903072 | 11 | 65583066 | OVOL1,SNX32 | intergenic | Impute | G | 0.53 | 0.53 | 0.51 | 1.05 | Michailidou K.Nat Genet.2015 |
| Breast | rs78540526 | 11 | 69331418 | LINC01488,CCND1 | intergenic | Impute | T | 0.07 | 0.07 | 0.09 | 1.34 | Michailidou K.Nat Genet.2015 |
| Breast | rs11820646 | 11 | 129461171 | BARX2,LINC01395 | intergenic | Impute | C | 0.63 | 0.60 | 0.62 | 1.05 | Michailidou K.Nat Genet.2015 |
| Breast | rs7297051 | 12 | 28174817 | PTHLH,CCDC91 | intergenic | Impute | C | 0.78 | 0.76 | 0.76 | 1.16 | Couch FJ.Nat Commun.2016 |
| Breast | rs17356907 | 12 | 96027759 | USP44,PGAM1P5 | intergenic | Genotype | A | 0.72 | 0.71 | 0.70 | 1.10 | Michailidou K.Nat Genet.2015 |
| Breast | rs1292011 | 12 | 115836522 | TBX3,MED13L | intergenic | Genotype | A | 0.60 | 0.58 | 0.58 | 1.09 | Michailidou K.Nat Genet.2015 |
| Breast | rs17181761 | 13 | 73811471 | KLF5,LINC00392 | intergenic | Impute | C | 0.31 | 0.32 | 0.33 | 1.09 | Couch FJ.Nat Commun.2016 |
| Breast | rs8002929 | 13 | 73964519 | KLF5,LINC00392 | intergenic | Genotype | A | 0.22 | 0.24 | 0.22 | 1.12 | Couch FJ.Nat Commun.2016 |
| Breast | rs2236007 | 14 | 37132769 | PAX9 | intronic | Impute | G | 0.80 | 0.79 | 0.78 | 1.08 | Michailidou K.Nat Genet.2015 |
| Breast | rs999737 | 14 | 69034682 | RAD51B | intronic | Genotype | C | 0.80 | 0.77 | 0.76 | 1.09 | Michailidou K.Nat Genet.2015 |
| Breast | rs941764 | 14 | 91841069 | CCDC88C | intronic | Impute | G | 0.36 | 0.34 | 0.35 | 1.07 | Michailidou K.Nat Genet.2015 |
| Breast | rs11627032 | 14 | 93104072 | RIN3 | intronic | Genotype | T | 0.76 | 0.74 | 0.75 | 1.06 | Michailidou K.Nat Genet.2015 |
| Breast | rs8051542 | 16 | 52534167 | TOX3 | intronic | Genotype | T | 0.44 | 0.44 | 0.45 | 1.09 | Easton DF.Nature.2007(11) |
| Breast | rs3803662 | 16 | 52586341 | CASC16 | ncRNA_exonic | Genotype | A | 0.30 | 0.27 | 0.28 | 1.24 | Michailidou K.Nat Genet.2015 |
| Breast | rs17817449 | 16 | 53813367 | FTO | intronic | Impute | T | 0.60 | 0.59 | 0.58 | 1.08 | Michailidou K.Nat Genet.2015 |
| Breast | rs13329835 | 16 | 80650805 | CDYL2 | intronic | Genotype | G | 0.23 | 0.22 | 0.23 | 1.08 | Michailidou K.Nat Genet.2015 |
| Breast | rs6504950 | 17 | 53056471 | STXBP4 | intronic | Impute | G | 0.74 | 0.73 | 0.73 | 1.06 | Michailidou K.Nat Genet.2015 |
| Breast | rs745570 | 17 | 77781725 | CBX8,CBX4 | intergenic | Impute | A | 0.50 | 0.50 | 0.49 | 1.05 | Michailidou K.Nat Genet.2015 |
| Breast | rs1436904 | 18 | 24570667 | CHST9 | intronic | Genotype | T | 0.61 | 0.60 | 0.61 | 1.04 | Michailidou K.Nat Genet.2015 |
| Breast | rs6507583 | 18 | 42399590 | SETBP1 | intronic | Impute | A | 0.94 | 0.93 | 0.94 | 1.10 | Michailidou K.Nat Genet.2015 |
| Breast | rs56069439 | 19 | 17393925 | ANKLE1 | intronic | Impute | A | 0.31 | 0.30 | 0.30 | 1.16 | Couch FJ.Nat Commun.2016 |
| Breast | rs4808801 | 19 | 18571141 | ELL | intronic | Genotype | A | 0.68 | 0.66 | 0.65 | 1.08 | Michailidou K.Nat Genet.2015 |
| Breast | rs3760982 | 19 | 44286513 | KCNN4,LYPD5 | intergenic | Genotype | A | 0.48 | 0.47 | 0.46 | 1.05 | Michailidou K.Nat Genet.2015 |
| Breast | rs6001930 | 22 | 40876234 | MKL1 | intronic | Impute | C | 0.12 | 0.11 | 0.12 | 1.12 | Michailidou K.Nat Genet.2015 |
| Colorectal | rs10911251 | 1 | 183081194 | LAMC1 | intronic | Impute | A | 0.58 | 0.56 | 0.57 | 1.07 | Schmit SL.J Natl Cancer Inst.2018(12) |
| Colorectal | rs6691170 | 1 | 222045446 | LOC101929771,HHIPL2 | intergenic | Genotype | T | 0.37 | 0.37 | 0.37 | 1.06 | Houlston RS.Nat Genet.2010(13) |
| Colorectal | rs6687758 | 1 | 222164948 | LOC101929771,HHIPL2 | intergenic | Genotype | G | 0.22 | 0.20 | 0.21 | 1.09 | Schmit SL.J Natl Cancer Inst.2018 |
| Colorectal | rs11903757 | 2 | 192587204 | LOC105747689,SDPR | intergenic | Impute | C | 0.15 | 0.15 | 0.16 | 1.16 | Peters U.Gastroenterology.2012(14) |
| Colorectal | rs992157 | 2 | 219154781 | PNKD,TMBIM1 | intronic | Genotype | G | 0.45 | 0.44 | 0.43 | 1.10 | Orlando G.Hum Mol Genet.2016(15) |
| Colorectal | rs1370821 | 4 | 94943383 | ATOH1,LOC101929210 | intergenic | Impute | T | 0.39 | 0.41 | 0.40 | 1.07 | Schmit SL.J Natl Cancer Inst.2018 |
| Colorectal | rs6906359 | 6 | 35528378 | TULP1,FKBP5 | intergenic | Impute | C | 0.91 | 0.88 | 0.89 | 1.11 | Schmit SL.J Natl Cancer Inst.2018 |
| Colorectal | rs1321311 | 6 | 36622900 | MIR3925,PANDAR | intergenic | Genotype | A | 0.26 | 0.24 | 0.24 | 1.10 | Dunlop MG.Nat Genet.2012(16) |
| Colorectal | rs62404968 | 6 | 55714314 | BMP5 | intronic | Impute | C | 0.76 | 0.76 | 0.75 | 1.09 | Schmit SL.J Natl Cancer Inst.2018 |
| Colorectal | rs16892766 | 8 | 117630683 | LINC00536,EIF3H | intergenic | Genotype | C | 0.08 | 0.08 | 0.08 | 1.27 | Tomlinson IP.Nat Genet.2008(17) |
| Colorectal | rs6983267 | 8 | 128413305 | CCAT2 | ncRNA_exonic | Genotype | G | 0.56 | 0.51 | 0.51 | 1.27 | Tomlinson I.Nat Genet.2007(18) |
| Colorectal | rs10994860 | 10 | 52645424 | A1CF | UTR5 | Impute | C | 0.84 | 0.83 | 0.82 | 1.09 | Pattaro C.Nat Commun.2016 |
| Colorectal | rs1035209 | 10 | 101345366 | NKX2-3,SLC25A28 | intergenic | Genotype | T | 0.19 | 0.19 | 0.20 | 1.09 | Schmit SL.J Natl Cancer Inst.2018 |
| Colorectal | rs1535 | 11 | 61597972 | FADS2 | intronic | Genotype | A | 0.70 | 0.66 | 0.66 | 1.07 | Schmit SL.J Natl Cancer Inst.2018 |
| Colorectal | rs3824999 | 11 | 74345550 | POLD3 | intronic | Genotype | G | 0.52 | 0.51 | 0.50 | 1.08 | Dunlop MG.Nat Genet.2012 |
| Colorectal | rs3802842 | 11 | 111171709 | COLCA2 | intronic | Genotype | C | 0.30 | 0.28 | 0.28 | 1.11 | Tenesa A.Nat Genet.2008(19) |
| Colorectal | rs7136702 | 12 | 50880216 | LARP4,DIP2B | intergenic | Genotype | T | 0.33 | 0.33 | 0.34 | 1.06 | Houlston RS.Nat Genet.2010 |
| Colorectal | rs11169552 | 12 | 51155663 | DIP2B,ATF1 | intergenic | Genotype | C | 0.74 | 0.73 | 0.71 | 1.09 | Houlston RS.Nat Genet.2010 |
| Colorectal | rs3184504 | 12 | 111884608 | SH2B3 | exonic | Genotype | C | 0.52 | 0.51 | 0.52 | 1.09 | Schumacher FR.Nat Commun.2015(20) |
| Colorectal | rs73208120 | 12 | 117747590 | NOS1 | intronic | Impute | G | 0.08 | 0.08 | 0.09 | 1.16 | Schumacher FR.Nat Commun.2015 |
| Colorectal | rs1957636 | 14 | 54560018 | BMP4,CDKN3 | intergenic | Genotype | T | 0.40 | 0.40 | 0.39 | 1.08 | Tomlinson IP.PLoS Genet.2011(21) |
| Colorectal | rs17094983 | 14 | 59189361 | DACT1,LINC01500 | intergenic | Impute | G | 0.88 | 0.88 | 0.87 | 1.15 | Mathieu Lemire.Hum Genet.2015(22) |
| Colorectal | rs4779584 | 15 | 32994756 | SCG5,GREM1 | intergenic | Genotype | C | 0.80 | 0.80 | 0.80 | 1.18 | Peters U.Hum Genet.2011(23) |
| Colorectal | rs1862748 | 16 | 68832943 | CDH1 | intronic | Genotype | C | 0.70 | 0.69 | 0.70 | 1.10 | Houlston RS.Nat Genet.2010 |
| Colorectal | rs4939827 | 18 | 46453463 | SMAD7 | intronic | Genotype | T | 0.58 | 0.51 | 0.52 | 1.20 | Tenesa A.Nat Genet.2008 |
| Colorectal | rs10411210 | 19 | 33532300 | RHPN2 | intronic | Genotype | C | 0.92 | 0.90 | 0.90 | 1.15 | Houlston RS.Nat Genet.2010 |
| Colorectal | rs355527 | 20 | 6388068 | FERMT1,CASC20 | intergenic | Impute | T | 0.33 | 0.32 | 0.32 | 1.12 | Houlston RS.Nat Genet.2010 |
| Colorectal | rs8124813 | 20 | 42105481 | SRSF6,L3MBTL1 | intergenic | Genotype | G | 0.35 | 0.34 | 0.35 | 1.09 | Schumacher FR.Nat Commun.2015 |
| Colorectal | rs1810502 | 20 | 49057488 | LINC01271,PTPN1 | intergenic | Impute | C | 0.57 | 0.56 | 0.55 | 1.08 | Schmit SL.J Natl Cancer Inst.2018 |
| Colorectal | rs4925386 | 20 | 60921044 | LAMA5 | intronic | Genotype | C | 0.70 | 0.69 | 0.72 | 1.08 | Houlston RS.Nat Genet.2010 |
| Glioma | rs12752552 | 1 | 65229299 | RAVER2 | intronic | Impute | T | 0.88 | 0.87 | 0.87 | 1.18 | Melin BS.Nat Genet.2017(24) |
| Glioma | rs4252707 | 1 | 204508147 | MDM4 | intronic | Impute | A | 0.21 | 0.19 | 0.20 | 1.19 | Melin BS.Nat Genet.2017 |
| Glioma | rs7572263 | 2 | 209051586 | C2orf80 | intronic | Genotype | A | 0.79 | 0.77 | 0.78 | 1.20 | Melin BS.Nat Genet.2017 |
| Glioma | rs11706832 | 3 | 66502981 | LRIG1 | intronic | Genotype | C | 0.51 | 0.48 | 0.45 | 1.15 | Melin BS.Nat Genet.2017 |
| Glioma | rs2736100 | 5 | 1286516 | TERT | intronic | Genotype | C | 0.58 | 0.51 | 0.50 | 1.29 | Melin BS.Nat Genet.2017 |
| Glioma | rs11979158 | 7 | 55159349 | EGFR | intronic | Genotype | A | 0.86 | 0.83 | 0.83 | 1.24 | Melin BS.Nat Genet.2017 |
| Glioma | rs4295627 | 8 | 130685457 | CCDC26 | ncRNA_intronic | Genotype | G | 0.22 | 0.18 | 0.18 | 1.36 | Shete S.Nat Genet.2009(25) |
| Glioma | rs634537 | 9 | 22032152 | CDKN2B-AS1 | ncRNA_intronic | Impute | G | 0.44 | 0.42 | 0.42 | 1.30 | Melin BS.Nat Genet.2017 |
| Glioma | rs11598018 | 10 | 105661315 | OBFC1 | intronic | Impute | C | 0.52 | 0.51 | 0.50 | 1.14 | Melin BS.Nat Genet.2017 |
| Glioma | rs11233250 | 11 | 82397014 | MIR4300HG,FAM181B | intergenic | Impute | C | 0.90 | 0.88 | 0.88 | 1.24 | Melin BS.Nat Genet.2017 |
| Glioma | rs7107785 | 11 | 95747337 | MAML2 | intronic | Impute | T | 0.51 | 0.49 | 0.49 | 1.16 | Melin BS.Nat Genet.2017 |
| Glioma | rs498872 | 11 | 118477367 | PHLDB1 | UTR5 | Genotype | A | 0.35 | 0.31 | 0.32 | 1.14 | Melin BS.Nat Genet.2017 |
| Glioma | rs12230172 | 12 | 76242675 | KRR1,PHLDA1 | intergenic | Impute | G | 0.56 | 0.53 | 0.53 | 1.23 | Kinnersley B.Nat Commun.2015(26) |
| Glioma | rs3851634 | 12 | 106812902 | POLR3B | intronic | Genotype | T | 0.72 | 0.71 | 0.71 | 1.23 | Kinnersley B.Nat Commun.2015 |
| Glioma | rs10131032 | 14 | 33250081 | AKAP6 | intronic | Genotype | G | 0.95 | 0.92 | 0.92 | 1.33 | Melin BS.Nat Genet.2017 |
| Glioma | rs1801591 | 15 | 76578762 | ETFA | exonic | Genotype | A | 0.10 | 0.09 | 0.10 | 1.33 | Melin BS.Nat Genet.2017 |
| Glioma | rs3751667 | 16 | 1004554 | LMF1 | exonic | Genotype | T | 0.26 | 0.23 | 0.23 | 1.14 | Melin BS.Nat Genet.2017 |
| Glioma | rs10852606 | 16 | 50128872 | HEATR3 | intronic | Impute | C | 0.74 | 0.72 | 0.74 | 1.14 | Melin BS.Nat Genet.2017 |
| Glioma | rs2297440 | 20 | 62312299 | RTEL1-TNFRSF6B | ncRNA_intronic | Genotype | C | 0.85 | 0.77 | 0.79 | 1.36 | Melin BS.Nat Genet.2017 |
| Lung | rs13314271 | 3 | 189357602 | TP63 | intronic | Impute | T | 0.52 | 0.50 | 0.49 | 1.13 | Wang Y.Nat Genet.2014(27) |
| Lung | rs9258375 | 6 | 29752808 | IFITM4P,HCG4 | intergenic | Impute | G | 0.08 | 0.08 | 0.10 | 1.25 | Fehringer G.Cancer Res.2016(28) |
| Lung | rs66759488 | 15 | 47577451 | SQRDL,SEMA6D | intergenic | Impute | A | 0.40 | 0.39 | 0.36 | 1.07 | McKay JD.Nat Genet.2017(29) |
| Lung | rs4887053 | 15 | 78712699 | CRABP1,IREB2 | intergenic | Genotype | C | 0.80 | 0.79 | 0.78 | 1.25 | Wang Y.Nat Genet.2008(30) |
| Lung | rs1051730 | 15 | 78894339 | CHRNA3 | exonic | Genotype | A | 0.37 | 0.35 | 0.33 | 1.31 | Landi MT.Am J Hum Genet.2011(31) |
| Lung | rs6495309 | 15 | 78915245 | CHRNA3,CHRNB4 | intergenic | Genotype | T | 0.20 | 0.21 | 0.22 | 1.30 | Wang Y.Nat Genet.2008 |
| Ovarian | rs2072590 | 2 | 177042633 | HAGLR | ncRNA_exonic | Genotype | A | 0.34 | 0.32 | 0.31 | 1.11 | Pharoah PD.Nat Genet.2013(32) |
| Ovarian | rs7651446 | 3 | 156406997 | TIPARP | intronic | Genotype | T | 0.08 | 0.05 | 0.05 | 1.44 | Pharoah PD.Nat Genet.2013 |
| Ovarian | rs4691139 | 4 | 165908721 | TRIM61,TRIM60 | intergenic | Genotype | G | 0.48 | 0.47 | 0.45 | 1.20 | Couch FJ.PLoS Genet.2013(33) |
| Ovarian | rs2736100 | 5 | 1286516 | TERT | intronic | Genotype | C | 0.54 | 0.51 | 0.50 | 1.43 | Bojesen SE.Nat Genet.2013(34) |
| Ovarian | rs11782652 | 8 | 82653644 | CHMP4C | intronic | Genotype | G | 0.10 | 0.07 | 0.07 | 1.19 | Pharoah PD.Nat Genet.2013 |
| Ovarian | rs10098821 | 8 | 129559228 | LINC00824 | ncRNA_intronic | Genotype | C | 0.90 | 0.89 | 0.90 | 1.21 | Goode EL.Nat Genet.2010(35) |
| Ovarian | rs635634 | 9 | 136155000 | ABO,SURF6 | intergenic | Impute | T | 0.20 | 0.21 | 0.20 | 1.12 | Kuchenbaecker KB.Nat Genet.2015(36) |
| Ovarian | rs183211 | 17 | 44788310 | NSF | intronic | Genotype | A | 0.27 | 0.23 | 0.22 | 1.25 | Couch FJ.PLoS Genet.2013 |
| Ovarian | rs9303542 | 17 | 46411500 | SKAP1 | intronic | Impute | G | 0.31 | 0.27 | 0.26 | 1.12 | Pharoah PD.Nat Genet.2013 |
| Ovarian | rs8170 | 19 | 17389704 | BABAM1 | exonic | Genotype | A | 0.20 | 0.19 | 0.20 | 1.18 | Michailidou K.Nat Genet.2015 |
| Pancreatic | rs11655237 | 17 | 70400166 | LINC00673 | ncRNA_exonic | Genotype | T | 0.15 | 0.11 | 0.12 | 1.26 | Childs EJ.Nat Genet.2015(37) |
| Pancreatic | rs6971499 | 7 | 130680521 | LINC-PINT | ncRNA_intronic | Impute | T | 0.88 | 0.85 | 0.85 | 1.27 | Wolpin BM.Nat Genet.2014(38) |
| Pancreatic | rs9581943 | 13 | 28493997 | PDX1-AS1 | ncRNA_intronic | Impute | A | 0.47 | 0.41 | 0.41 | 1.15 | Wolpin BM.Nat Genet.2014 |
| Pancreatic | rs9543325 | 13 | 73916628 | KLF5,LINC00392 | intergenic | Genotype | C | 0.42 | 0.36 | 0.36 | 1.26 | Petersen GM.Nat Genet.2010(39) |
| Pancreatic | rs401681 | 5 | 1322087 | CLPTM1L | intronic | Genotype | T | 0.50 | 0.44 | 0.43 | 1.20 | Childs EJ.Nat Genet.2015 |
| Pancreatic | rs16986825 | 22 | 29300306 | ZNRF3 | intronic | Impute | T | 0.17 | 0.15 | 0.15 | 1.18 | Wolpin BM.Nat Genet.2014 |
| Pancreatic | rs17688601 | 7 | 40866663 | SUGCT | intronic | Genotype | C | 0.75 | 0.74 | 0.72 | 1.14 | Childs EJ.Nat Genet.2015 |
| Pancreatic | rs1486134 | 2 | 67639769 | ETAA1,LOC101927701 | intergenic | Genotype | G | 0.30 | 0.29 | 0.31 | 1.14 | Childs EJ.Nat Genet.2015 |
| Pancreatic | rs3790844 | 1 | 200007432 | NR5A2 | intronic | Genotype | A | 0.76 | 0.77 | 0.74 | 1.30 | Petersen GM.Nat Genet.2010 |
| Prostate | rs636291 | 1 | 10556097 | PEX14 | intronic | Impute | A | 0.70 | 0.68 | 0.69 | 1.05 | Olama.Nat Genet.2014(40) |
| Prostate | rs4245739 | 1 | 204518842 | MDM4 | UTR3 | Impute | A | 0.75 | 0.73 | 0.76 | 1.13 | Schumacher FR.Nat Genet.2018(41) |
| Prostate | rs9287719 | 2 | 10710730 | NOL10 | downstream | Impute | C | 0.50 | 0.47 | 0.45 | 1.13 | Schumacher FR.Nat Genet.2018 |
| Prostate | rs9306895 | 2 | 20878153 | GDF7,LDAH | intergenic | Impute | C | 0.41 | 0.36 | 0.38 | 1.26 | Schumacher FR.Nat Genet.2018 |
| Prostate | rs721048 | 2 | 63131731 | EHBP1 | intronic | Impute | A | 0.19 | 0.18 | 0.17 | 1.06 | Schumacher FR.Nat Genet.2018 |
| Prostate | rs10187424 | 2 | 85794297 | GGCX,VAMP8 | intergenic | Genotype | T | 0.62 | 0.58 | 0.59 | 1.18 | Schumacher FR.Nat Genet.2018 |
| Prostate | rs12621278 | 2 | 173311553 | ITGA6 | intronic | Genotype | A | 0.95 | 0.94 | 0.94 | 1.36 | Schumacher FR.Nat Genet.2018 |
| Prostate | rs34925593 | 2 | 174234547 | CDCA7 | downstream | Impute | C | 0.48 | 0.49 | 0.50 | 0.97 | Schumacher FR.Nat Genet.2018 |
| Prostate | rs7584330 | 2 | 238387228 | COL6A3,MLPH | intergenic | Genotype | G | 0.26 | 0.24 | 0.22 | 1.14 | Kote-Jarai Z.Nat Genet.2011(42) |
| Prostate | rs3771570 | 2 | 242382864 | FARP2 | intronic | Genotype | T | 0.16 | 0.15 | 0.15 | 1.07 | Schumacher FR.Nat Genet.2018 |
| Prostate | rs2660753 | 3 | 87110674 | VGLL3,LINC00506 | intergenic | Genotype | T | 0.12 | 0.11 | 0.12 | 1.08 | Schumacher FR.Nat Genet.2018 |
| Prostate | rs7611694 | 3 | 113275624 | SIDT1 | intronic | Genotype | A | 0.60 | 0.59 | 0.57 | 1.07 | Schumacher FR.Nat Genet.2018 |
| Prostate | rs10934853 | 3 | 128038373 | EEFSEC | intronic | Genotype | A | 0.28 | 0.28 | 0.29 | 1.03 | Schumacher FR.Nat Genet.2018 |
| Prostate | rs6763931 | 3 | 141102833 | ZBTB38 | intronic | Genotype | A | 0.45 | 0.43 | 0.43 | 1.05 | Kote-Jarai Z.Nat Genet.2011 |
| Prostate | rs1894292 | 4 | 74349158 | AFM | intronic | Genotype | G | 0.51 | 0.52 | 0.53 | 0.95 | Schumacher FR.Nat Genet.2018 |
| Prostate | rs17021918 | 4 | 95562877 | PDLIM5 | intronic | Genotype | C | 0.69 | 0.65 | 0.65 | 1.17 | Schumacher FR.Nat Genet.2018 |
| Prostate | rs7679673 | 4 | 106061534 | LOC101929468,TET2 | intergenic | Impute | C | 0.63 | 0.60 | 0.58 | 1.10 | Schumacher FR.Nat Genet.2018 |
| Prostate | rs2121875 | 5 | 44365545 | FGF10 | intronic | Genotype | C | 0.36 | 0.33 | 0.32 | 1.15 | Schumacher FR.Nat Genet.2018 |
| Prostate | rs76551843 | 5 | 169172133 | DOCK2 | intronic | Impute | A | 1.00 | 0.99 | 0.99 | 1.85 | Schumacher FR.Nat Genet.2018 |
| Prostate | rs6869841 | 5 | 172939426 | MIR8056,LOC285593 | intergenic | Impute | T | 0.23 | 0.21 | 0.21 | 1.08 | Eeles RA.Nat Genet.2013(43) |
| Prostate | rs4976790 | 5 | 177968915 | COL23A1 | intronic | Genotype | T | 0.12 | 0.12 | 0.11 | 0.99 | Schumacher FR.Nat Genet.2018 |
| Prostate | rs4713266 | 6 | 11219030 | NEDD9 | intronic | Impute | C | 0.54 | 0.51 | 0.53 | 1.12 | Schumacher FR.Nat Genet.2018 |
| Prostate | rs12665339 | 6 | 30601232 | ATAT1 | intronic | Genotype | G | 0.16 | 0.16 | 0.17 | 0.97 | Schumacher FR.Nat Genet.2018 |
| Prostate | rs130067 | 6 | 31118511 | CCHCR1 | exonic | Genotype | G | 0.21 | 0.20 | 0.20 | 1.04 | Kote-Jarai Z.Nat Genet.2011 |
| Prostate | rs9296068 | 6 | 32988695 | HLA-DOA,HLA-DPA1 | intergenic | Genotype | T | 0.66 | 0.66 | 0.65 | 0.97 | Schumacher FR.Nat Genet.2018 |
| Prostate | rs9469899 | 6 | 34793124 | UHRF1BP1 | intronic | Impute | A | 0.35 | 0.36 | 0.37 | 0.97 | Schumacher FR.Nat Genet.2018 |
| Prostate | rs1983891 | 6 | 41536427 | FOXP4 | intronic | Genotype | T | 0.28 | 0.27 | 0.29 | 1.04 | Schumacher FR.Nat Genet.2018 |
| Prostate | rs9443189 | 6 | 76495882 | MYO6 | intronic | Impute | A | 0.87 | 0.87 | 0.87 | 1.04 | Schumacher FR.Nat Genet.2018 |
| Prostate | rs2273669 | 6 | 109285189 | ARMC2 | intronic | Genotype | G | 0.15 | 0.14 | 0.15 | 1.05 | Schumacher FR.Nat Genet.2018 |
| Prostate | rs339331 | 6 | 117210052 | RFX6 | intronic | Genotype | T | 0.71 | 0.69 | 0.68 | 1.10 | Schumacher FR.Nat Genet.2018 |
| Prostate | rs1933488 | 6 | 153441079 | RGS17 | intronic | Genotype | A | 0.58 | 0.58 | 0.56 | 1.00 | Schumacher FR.Nat Genet.2018 |
| Prostate | rs9364554 | 6 | 160833664 | SLC22A3 | intronic | Genotype | T | 0.31 | 0.29 | 0.27 | 1.12 | Schumacher FR.Nat Genet.2018 |
| Prostate | rs138004030 | 6 | 170475879 | LOC102724511 | ncRNA_exonic | Impute | G | 0.92 | 0.93 | 0.94 | 0.96 | Schumacher FR.Nat Genet.2018 |
| Prostate | rs12155172 | 7 | 20994491 | LINC01162 | ncRNA_intronic | Impute | A | 0.23 | 0.22 | 0.24 | 1.07 | Schumacher FR.Nat Genet.2018 |
| Prostate | rs10486567 | 7 | 27976563 | JAZF1 | intronic | Genotype | G | 0.77 | 0.77 | 0.77 | 1.00 | Schumacher FR.Nat Genet.2018 |
| Prostate | rs17621345 | 7 | 40875192 | SUGCT | intronic | Impute | A | 0.75 | 0.73 | 0.74 | 1.08 | Schumacher FR.Nat Genet.2018 |
| Prostate | rs56232506 | 7 | 47437244 | TNS3 | intronic | Impute | A | 0.49 | 0.46 | 0.47 | 1.09 | Schumacher FR.Nat Genet.2018 |
| Prostate | rs6465657 | 7 | 97816327 | LMTK2 | intronic | Genotype | C | 0.48 | 0.47 | 0.48 | 1.05 | Schumacher FR.Nat Genet.2018 |
| Prostate | rs2928679 | 8 | 23438975 | SLC25A37,NKX3-1 | intergenic | Genotype | A | 0.44 | 0.45 | 0.43 | 0.95 | Schumacher FR.Nat Genet.2018 |
| Prostate | rs1512268 | 8 | 23526463 | SLC25A37,NKX3-1 | intergenic | Genotype | T | 0.45 | 0.43 | 0.43 | 1.09 | Schumacher FR.Nat Genet.2018 |
| Prostate | rs11135910 | 8 | 25892142 | EBF2 | intronic | Genotype | T | 0.15 | 0.16 | 0.15 | 0.94 | Schumacher FR.Nat Genet.2018 |
| Prostate | rs10086908 | 8 | 128011937 | FAM84B,PCAT1 | intergenic | Genotype | T | 0.74 | 0.70 | 0.71 | 1.20 | Schumacher FR.Nat Genet.2018 |
| Prostate | rs13252298 | 8 | 128095156 | PRNCR1 | ncRNA_exonic | Genotype | A | 0.75 | 0.71 | 0.70 | 1.23 | Al Olama AA.Nat Genet.2009(44) |
| Prostate | rs16901979 | 8 | 128124916 | PRNCR1,CASC19 | intergenic | Impute | A | 0.05 | 0.03 | 0.03 | 1.32 | Schumacher FR.Nat Genet.2018 |
| Prostate | rs16902094 | 8 | 128320346 | CASC21,CASC8 | ncRNA_intronic | Impute | G | 0.14 | 0.13 | 0.16 | 1.11 | Gudmundsson J.Nat Genet.2009(45) |
| Prostate | rs445114 | 8 | 128323181 | CASC21,CASC8 | ncRNA_intronic | Impute | T | 0.67 | 0.65 | 0.64 | 1.10 | Gudmundsson J.Nat Genet.2009 |
| Prostate | rs16902104 | 8 | 128340908 | CASC21,CASC8 | ncRNA_intronic | Genotype | T | 0.15 | 0.14 | 0.16 | 1.14 | Hoffmann TJ.Cancer Discov.2015(46) |
| Prostate | rs6983267 | 8 | 128413305 | CCAT2 | ncRNA_exonic | Genotype | G | 0.57 | 0.51 | 0.51 | 1.27 | Schumacher FR.Nat Genet.2018 |
| Prostate | rs7837688 | 8 | 128539360 | CASC8,CASC11 | intergenic | Genotype | T | 0.14 | 0.10 | 0.11 | 1.41 | Yeager M.Nat Genet.2007(47) |
| Prostate | rs1048169 | 9 | 19055965 | HAUS6 | UTR3 | Impute | C | 0.39 | 0.39 | 0.38 | 1.01 | Schumacher FR.Nat Genet.2018 |
| Prostate | rs1182 | 9 | 132576060 | TOR1A | UTR3 | Impute | A | 0.24 | 0.22 | 0.22 | 1.15 | Schumacher FR.Nat Genet.2018 |
| Prostate | rs1935581 | 10 | 90195149 | RNLS | intronic | Genotype | C | 0.63 | 0.63 | 0.62 | 0.98 | Schumacher FR.Nat Genet.2018 |
| Prostate | rs3850699 | 10 | 104414221 | TRIM8 | intronic | Genotype | A | 0.72 | 0.70 | 0.68 | 1.10 | Schumacher FR.Nat Genet.2018 |
| Prostate | rs4962416 | 10 | 126696872 | CTBP2 | intronic | Genotype | C | 0.28 | 0.28 | 0.27 | 0.97 | Schumacher FR.Nat Genet.2018 |
| Prostate | rs1881502 | 11 | 1507512 | MOB2 | intronic | Impute | T | 0.20 | 0.19 | 0.20 | 1.08 | Schumacher FR.Nat Genet.2018 |
| Prostate | rs61890184 | 11 | 7547587 | PPFIBP2 | intronic | Impute | A | 0.13 | 0.11 | 0.12 | 1.18 | Schumacher FR.Nat Genet.2018 |
| Prostate | rs7931342 | 11 | 68994497 | LOC338694,MYEOV | intergenic | Genotype | G | 0.56 | 0.51 | 0.49 | 1.22 | Schumacher FR.Nat Genet.2018 |
| Prostate | rs138466039 | 11 | 125054793 | PKNOX2 | intronic | Impute | T | 0.01 | 0.01 | 0.01 | 1.04 | Schumacher FR.Nat Genet.2018 |
| Prostate | rs80130819 | 12 | 48419618 | COL2A1,SENP1 | intergenic | Impute | A | 0.92 | 0.92 | 0.91 | 1.06 | Schumacher FR.Nat Genet.2018 |
| Prostate | rs902774 | 12 | 53273904 | KRT78,KRT8 | intergenic | Genotype | A | 0.18 | 0.15 | 0.15 | 1.26 | Schumacher FR.Nat Genet.2018 |
| Prostate | rs7968403 | 12 | 65012824 | RASSF3 | intronic | Impute | T | 0.66 | 0.65 | 0.65 | 1.06 | Schumacher FR.Nat Genet.2018 |
| Prostate | rs1270884 | 12 | 114685571 | RBM19,TBX5 | intergenic | Impute | A | 0.50 | 0.49 | 0.49 | 1.04 | Schumacher FR.Nat Genet.2018 |
| Prostate | rs8008270 | 14 | 53372330 | FERMT2 | intronic | Genotype | C | 0.80 | 0.81 | 0.81 | 0.97 | Schumacher FR.Nat Genet.2018 |
| Prostate | rs7141529 | 14 | 69126744 | RAD51B,ZFP36L1 | intergenic | Genotype | C | 0.49 | 0.50 | 0.48 | 0.94 | Schumacher FR.Nat Genet.2018 |
| Prostate | rs684232 | 17 | 618965 | VPS53 | upstream | Impute | C | 0.37 | 0.36 | 0.36 | 1.04 | Schumacher FR.Nat Genet.2018 |
| Prostate | rs28441558 | 17 | 7803118 | CHD3 | intronic | Impute | C | 0.06 | 0.05 | 0.06 | 1.21 | Schumacher FR.Nat Genet.2018 |
| Prostate | rs11649743 | 17 | 36074979 | HNF1B | intronic | Genotype | G | 0.86 | 0.81 | 0.82 | 1.43 | Schumacher FR.Nat Genet.2018 |
| Prostate | rs4430796 | 17 | 36098040 | HNF1B | intronic | Genotype | A | 0.56 | 0.52 | 0.55 | 1.18 | Schumacher FR.Nat Genet.2018 |
| Prostate | rs11650494 | 17 | 47345186 | FLJ40194,MIR6129 | intergenic | Impute | A | 0.09 | 0.08 | 0.07 | 1.19 | Schumacher FR.Nat Genet.2018 |
| Prostate | rs2680708 | 17 | 56456120 | RNF43 | intronic | Impute | G | 0.60 | 0.62 | 0.60 | 0.92 | Schumacher FR.Nat Genet.2018 |
| Prostate | rs1859962 | 17 | 69108753 | CASC17 | ncRNA_intronic | Genotype | G | 0.56 | 0.48 | 0.49 | 1.38 | Schumacher FR.Nat Genet.2018 |
| Prostate | rs12956892 | 18 | 56746315 | OACYLP,SEC11C | intergenic | Impute | T | 0.31 | 0.30 | 0.30 | 1.04 | Schumacher FR.Nat Genet.2018 |
| Prostate | rs10460109 | 18 | 73036165 | TSHZ1,SMIM21 | intergenic | Impute | T | 0.44 | 0.42 | 0.43 | 1.07 | Schumacher FR.Nat Genet.2018 |
| Prostate | rs11666569 | 19 | 17214073 | MYO9B | intronic | Impute | C | 0.69 | 0.70 | 0.72 | 0.94 | Schumacher FR.Nat Genet.2018 |
| Prostate | rs2735839 | 19 | 51364623 | KLK3 | downstream | Genotype | G | 0.85 | 0.86 | 0.87 | 0.94 | Schumacher FR.Nat Genet.2018 |
| Prostate | rs12480328 | 20 | 49527922 | ADNP | intronic | Impute | T | 0.94 | 0.93 | 0.93 | 1.17 | Schumacher FR.Nat Genet.2018 |
| Prostate | rs6062509 | 20 | 62362563 | ZGPAT | intronic | Impute | T | 0.68 | 0.70 | 0.70 | 0.92 | Schumacher FR.Nat Genet.2018 |
| Prostate | rs9625483 | 22 | 28888939 | TTC28 | intronic | Impute | A | 0.02 | 0.02 | 0.04 | 1.00 | Schumacher FR.Nat Genet.2018 |
| Prostate | rs5759167 | 22 | 43500212 | TTLL1,BIK | intergenic | Genotype | G | 0.53 | 0.50 | 0.50 | 1.12 | Schumacher FR.Nat Genet.2018 |
| Renal | rs4381241 | 1 | 50907438 | FAF1 | intronic | Impute | C | 0.50 | 0.46 | 0.44 | 1.11 | Ghislaine Scelo.Nat Commun.2017(48) |
| Renal | rs3845536 | 1 | 165650787 | ALDH9A1 | intronic | Impute | C | 0.68 | 0.66 | 0.64 | 1.21 | Henrion MY.Plos One.2015(49) |
| Renal | rs7579899 | 2 | 46537604 | EPAS1 | intronic | Genotype | A | 0.39 | 0.40 | 0.40 | 1.15 | Purdue MP.Nat Genet.2010(50) |
| Renal | rs12105918 | 2 | 145208193 | ZEB2 | intronic | Genotype | C | 0.08 | 0.06 | 0.05 | 1.29 | Henrion M.Hum Mol Genet.2012(51) |
| Renal | rs10936602 | 3 | 169536637 | LRRC34,LRRIQ4 | intergenic | Impute | T | 0.76 | 0.74 | 0.73 | 1.06 | Ghislaine Scelo.Nat Commun.2017 |
| Renal | rs2241261 | 8 | 22876739 | RHOBTB2 | UTR3 | Impute | T | 0.53 | 0.53 | 0.51 | 1.10 | Ghislaine Scelo.Nat Commun.2017 |
| Renal | rs11813268 | 10 | 105682296 | OBFC1,SLK | intergenic | Impute | T | 0.20 | 0.16 | 0.16 | 1.12 | Ghislaine Scelo.Nat Commun.2017 |
| Renal | rs7105934 | 11 | 69239741 | MYEOV,LINC01488 | intergenic | Genotype | G | 0.96 | 0.92 | 0.93 | 1.45 | Purdue MP.Nat Genet.2010 |
| Renal | rs74911261 | 11 | 108357137 | KDELC2 | exonic | Impute | A | 0.02 | 0.02 | 0.02 | 1.41 | Ghislaine Scelo.Nat Commun.2017 |
| Renal | rs718314 | 12 | 26453283 | SSPN,ITPR2 | intergenic | Genotype | G | 0.31 | 0.25 | 0.27 | 1.19 | Wu X.Hum Mol Genet.2011(52) |
| Melanoma | rs7412746 | 1 | 150860471 | ARNT,SETDB1 | intergenic | Genotype | T | 0.55 | 0.55 | 0.55 | 1.09 | Ransohoff KJ.Oncotarget.2017(53) |
| Melanoma | rs6750047 | 2 | 38276549 | RMDN2 | intronic | Impute | A | 0.50 | 0.49 | 0.48 | 1.03 | Ransohoff KJ.Oncotarget.2017 |
| Melanoma | rs700635 | 2 | 202153225 | ALS2CR12 | UTR3 | Impute | A | 0.70 | 0.72 | 0.73 | 1.15 | Barrett JH.Nat Genet.2011(54) |
| Melanoma | rs401681 | 5 | 1322087 | CLPTM1L | intronic | Genotype | T | 0.47 | 0.44 | 0.43 | 1.20 | Barrett JH.Nat Genet.2011 |
| Melanoma | rs1636744 | 7 | 16984280 | AGR3,AHR | intergenic | Impute | T | 0.40 | 0.41 | 0.41 | 1.05 | Ransohoff KJ.Oncotarget.2017 |
| Melanoma | rs4636294 | 9 | 21747803 | MIR31HG,MTAP | intergenic | Genotype | A | 0.54 | 0.48 | 0.47 | 1.21 | Falchi M.Nat Genet.2009(55) |
| Melanoma | rs10739221 | 9 | 109060830 | TMEM38B,MIR8081 | intergenic | Impute | T | 0.25 | 0.23 | 0.25 | 1.12 | Ransohoff KJ.Oncotarget.2017 |
| Melanoma | rs2995264 | 10 | 105668843 | OBFC1 | intronic | Impute | G | 0.11 | 0.10 | 0.10 | 1.17 | Ransohoff KJ.Oncotarget.2017 |
| Melanoma | rs498136 | 11 | 69367118 | LINC01488,CCND1 | intergenic | Impute | A | 0.38 | 0.34 | 0.34 | 1.09 | Ransohoff KJ.Oncotarget.2017 |
| Melanoma | rs1847142 | 11 | 89021574 | TYR | intronic | Impute | A | 0.35 | 0.31 | 0.31 | 1.31 | Bishop DT.Nat Genet.2009(56) |
| Melanoma | rs1801516 | 11 | 108175462 | ATM | exonic | Genotype | G | 0.86 | 0.86 | 0.85 | 1.08 | Ransohoff KJ.Oncotarget.2017 |
| Melanoma | rs16953002 | 16 | 54114824 | FTO | intronic | Genotype | A | 0.19 | 0.17 | 0.18 | 1.03 | Ransohoff KJ.Oncotarget.2017 |
| Melanoma | rs7188458 | 16 | 89726484 | SPATA33 | intronic | Genotype | A | 0.48 | 0.45 | 0.45 | 1.30 | Bishop DT.Nat Genet.2009 |
| Melanoma | rs1805007 | 16 | 89986117 | MC1R | exonic | Impute | T | 0.10 | 0.07 | 0.07 | 1.50 | Nan H.Hum Mol Genet.2011(57) |
| Melanoma | rs17305657 | 20 | 31806588 | BPIFA3 | intronic | Genotype | C | 0.11 | 0.08 | 0.08 | 1.58 | Brown KM.Nat Genet.2008(58) |
| Melanoma | rs45430 | 21 | 42746081 | MX2 | intronic | Genotype | T | 0.67 | 0.61 | 0.61 | 1.12 | Ransohoff KJ.Oncotarget.2017 |
| Melanoma | rs6001027 | 22 | 38545619 | PLA2G6 | intronic | Genotype | A | 0.66 | 0.65 | 0.64 | 1.20 | Bishop DT.Nat Genet.2009 |
| Thyroid | rs12129938 | 1 | 233412561 | PCNXL2 | intronic | Impute | A | 0.78 | 0.77 | 0.78 | 1.32 | Gudmundsson J.Nat Commun.2017(59) |
| Thyroid | rs966423 | 2 | 218310340 | DIRC3 | ncRNA_intronic | Genotype | C | 0.47 | 0.42 | 0.41 | 1.34 | Gudmundsson J.Nat Genet.2012(60) |
| Thyroid | rs6793295 | 3 | 169518455 | LRRC34 | exonic | Genotype | T | 0.76 | 0.73 | 0.73 | 1.23 | Gudmundsson J.Nat Commun.2017 |
| Thyroid | rs7902587 | 10 | 105694301 | OBFC1,SLK | intergenic | Impute | T | 0.12 | 0.09 | 0.10 | 1.41 | Gudmundsson J.Nat Commun.2017 |
| Thyroid | rs944289 | 14 | 36649246 | PTCSC3,MBIP | intergenic | Genotype | T | 0.62 | 0.58 | 0.59 | 1.37 | Gudmundsson J.Nat Genet.2009(61) |
| Thyroid | rs7267944 | 20 | 37947434 | LOC339568,LINC01370 | intergenic | Impute | C | 0.18 | 0.18 | 0.18 | 1.39 | Figlioli G.J Clin Endocrinol Metab.2014(62) |

Abbreviations: SNP, single nucleotide polymorphism; CHR, chromosome; POS, position; RA, risk allele; RAF, risk allele frequency; OR, odds ratio.

^1^RAF is based on the genome Aggregation Database (gnomAD), Non-Finnish European (NFE) population.

^2^OR is based on the OncoArray: Prostate Cancer (dbGaP accession phs001391.v1.p1).

**References**

1. Figueroa JD, Ye Y, Siddiq A, Garcia-Closas M, Chatterjee N, Prokunina-Olsson L, et al. Genome-wide association study identifies multiple loci associated with bladder cancer risk. Hum Mol Genet. 2014;23(5):1387-98.

2. Wu X, Ye Y, Kiemeney LA, Sulem P, Rafnar T, Matullo G, et al. Genetic variation in the prostate stem cell antigen gene PSCA confers susceptibility to urinary bladder cancer. Nat Genet. 2009;41(9):991-5.

3. Rafnar T, Vermeulen SH, Sulem P, Thorleifsson G, Aben KK, Witjes JA, et al. European genome-wide association study identifies SLC14A1 as a new urinary bladder cancer susceptibility gene. Hum Mol Genet. 2011;20(21):4268-81.

4. Rafnar T, Sulem P, Thorleifsson G, Vermeulen SH, Helgason H, Saemundsdottir J, et al. Genome-wide association study yields variants at 20p12.2 that associate with urinary bladder cancer. Hum Mol Genet. 2014;23(20):5545-57.

5. Couch FJ, Kuchenbaecker KB, Michailidou K, Mendoza-Fandino GA, Nord S, Lilyquist J, et al. Identification of four novel susceptibility loci for oestrogen receptor negative breast cancer. Nat Commun. 2016;7:11375.

6. Michailidou K, Beesley J, Lindstrom S, Canisius S, Dennis J, Lush MJ, et al. Genome-wide association analysis of more than 120,000 individuals identifies 15 new susceptibility loci for breast cancer. Nat Genet. 2015;47(4):373-80.

7. Lin WY, Camp NJ, Ghoussaini M, Beesley J, Michailidou K, Hopper JL, et al. Identification and characterization of novel associations in the CASP8/ALS2CR12 region on chromosome 2 with breast cancer risk. Hum Mol Genet. 2015;24(1):285-98.

8. Michailidou K, Hall P, Gonzalez-Neira A, Ghoussaini M, Dennis J, Milne RL, et al. Large-scale genotyping identifies 41 new loci associated with breast cancer risk. Nat Genet. 2013;45(4):353-61, 61e1-2.

9. Gold B, Kirchhoff T, Stefanov S, Lautenberger J, Viale A, Garber J, et al. Genome-wide association study provides evidence for a breast cancer risk locus at 6q22.33. Proc Natl Acad Sci U S A. 2008;105(11):4340-5.

10. Fletcher O, Johnson N, Orr N, Hosking FJ, Gibson LJ, Walker K, et al. Novel breast cancer susceptibility locus at 9q31.2: results of a genome-wide association study. J Natl Cancer Inst. 2011;103(5):425-35.

11. Easton DF, Pooley KA, Dunning AM, Pharoah PD, Thompson D, Ballinger DG, et al. Genome-wide association study identifies novel breast cancer susceptibility loci. Nature. 2007;447(7148):1087-93.

12. Schmit SL, Edlund CK, Schumacher FR, Gong J, Harrison TA, Huyghe JR, et al. Novel Common Genetic Susceptibility Loci for Colorectal Cancer. J Natl Cancer Inst. 2018.

13. Houlston RS, Cheadle J, Dobbins SE, Tenesa A, Jones AM, Howarth K, et al. Meta-analysis of three genome-wide association studies identifies susceptibility loci for colorectal cancer at 1q41, 3q26.2, 12q13.13 and 20q13.33. Nat Genet. 2010;42(11):973-7.

14. Peters U, Jiao S, Schumacher FR, Hutter CM, Aragaki AK, Baron JA, et al. Identification of Genetic Susceptibility Loci for Colorectal Tumors in a Genome-Wide Meta-analysis. Gastroenterology. 2013;144(4):799-807 e24.

15. Orlando G, Law PJ, Palin K, Tuupanen S, Gylfe A, Hanninen UA, et al. Variation at 2q35 (PNKD and TMBIM1) influences colorectal cancer risk and identifies a pleiotropic effect with inflammatory bowel disease. Hum Mol Genet. 2016;25(11):2349-59.

16. Dunlop MG, Dobbins SE, Farrington SM, Jones AM, Palles C, Whiffin N, et al. Common variation near CDKN1A, POLD3 and SHROOM2 influences colorectal cancer risk. Nat Genet. 2012;44(7):770-6.

17. Tomlinson IP, Webb E, Carvajal-Carmona L, Broderick P, Howarth K, Pittman AM, et al. A genome-wide association study identifies colorectal cancer susceptibility loci on chromosomes 10p14 and 8q23.3. Nat Genet. 2008;40(5):623-30.

18. Tomlinson I, Webb E, Carvajal-Carmona L, Broderick P, Kemp Z, Spain S, et al. A genome-wide association scan of tag SNPs identifies a susceptibility variant for colorectal cancer at 8q24.21. Nat Genet. 2007;39(8):984-8.

19. Tenesa A, Farrington SM, Prendergast JG, Porteous ME, Walker M, Haq N, et al. Genome-wide association scan identifies a colorectal cancer susceptibility locus on 11q23 and replicates risk loci at 8q24 and 18q21. Nat Genet. 2008;40(5):631-7.

20. Schumacher FR, Schmit SL, Jiao S, Edlund CK, Wang H, Zhang B, et al. Genome-wide association study of colorectal cancer identifies six new susceptibility loci. Nat Commun. 2015;6:7138.

21. Tomlinson IP, Carvajal-Carmona LG, Dobbins SE, Tenesa A, Jones AM, Howarth K, et al. Multiple common susceptibility variants near BMP pathway loci GREM1, BMP4, and BMP2 explain part of the missing heritability of colorectal cancer. PLoS Genet. 2011;7(6):e1002105.

22. Lemire M, Qu C, Loo LWM, Zaidi SHE, Wang H, Berndt SI, et al. A genome-wide association study for colorectal cancer identifies a risk locus in 14q23.1. Hum Genet. 2015;134(11-12):1249-62.

23. Peters U, Hutter CM, Hsu L, Schumacher FR, Conti DV, Carlson CS, et al. Meta-analysis of new genome-wide association studies of colorectal cancer risk. Hum Genet. 2012;131(2):217-34.

24. Melin BS, Barnholtz-Sloan JS, Wrensch MR, Johansen C, Il'yasova D, Kinnersley B, et al. Genome-wide association study of glioma subtypes identifies specific differences in genetic susceptibility to glioblastoma and non-glioblastoma tumors. Nat Genet. 2017;49(5):789-94.

25. Shete S, Hosking FJ, Robertson LB, Dobbins SE, Sanson M, Malmer B, et al. Genome-wide association study identifies five susceptibility loci for glioma. Nat Genet. 2009;41(8):899-904.

26. Kinnersley B, Labussiere M, Holroyd A, Di Stefano AL, Broderick P, Vijayakrishnan J, et al. Genome-wide association study identifies multiple susceptibility loci for glioma. Nat Commun. 2015;6:8559.

27. Wang Y, McKay JD, Rafnar T, Wang Z, Timofeeva MN, Broderick P, et al. Rare variants of large effect in BRCA2 and CHEK2 affect risk of lung cancer. Nat Genet. 2014;46(7):736-41.

28. Fehringer G, Kraft P, Pharoah PD, Eeles RA, Chatterjee N, Schumacher FR, et al. Cross-Cancer Genome-Wide Analysis of Lung, Ovary, Breast, Prostate, and Colorectal Cancer Reveals Novel Pleiotropic Associations. Cancer Res. 2016;76(17):5103-14.

29. McKay JD, Hung RJ, Han Y, Zong X, Carreras-Torres R, Christiani DC, et al. Large-scale association analysis identifies new lung cancer susceptibility loci and heterogeneity in genetic susceptibility across histological subtypes. Nat Genet. 2017;49(7):1126-32.

30. Wang Y, Broderick P, Webb E, Wu X, Vijayakrishnan J, Matakidou A, et al. Common 5p15.33 and 6p21.33 variants influence lung cancer risk. Nat Genet. 2008;40(12):1407-9.

31. Landi MT, Chatterjee N, Yu K, Goldin LR, Goldstein AM, Rotunno M, et al. A genome-wide association study of lung cancer identifies a region of chromosome 5p15 associated with risk for adenocarcinoma. Am J Hum Genet. 2009;85(5):679-91.

32. Pharoah PD, Tsai YY, Ramus SJ, Phelan CM, Goode EL, Lawrenson K, et al. GWAS meta-analysis and replication identifies three new susceptibility loci for ovarian cancer. Nat Genet. 2013;45(4):362-70, 70e1-2.

33. Couch FJ, Wang X, McGuffog L, Lee A, Olswold C, Kuchenbaecker KB, et al. Genome-wide association study in BRCA1 mutation carriers identifies novel loci associated with breast and ovarian cancer risk. PLoS Genet. 2013;9(3):e1003212.

34. Bojesen SE, Pooley KA, Johnatty SE, Beesley J, Michailidou K, Tyrer JP, et al. Multiple independent variants at the TERT locus are associated with telomere length and risks of breast and ovarian cancer. Nat Genet. 2013;45(4):371-84, 84e1-2.

35. Goode EL, Chenevix-Trench G, Song H, Ramus SJ, Notaridou M, Lawrenson K, et al. A genome-wide association study identifies susceptibility loci for ovarian cancer at 2q31 and 8q24. Nat Genet. 2010;42(10):874-9.

36. Kuchenbaecker KB, Ramus SJ, Tyrer J, Lee A, Shen HC, Beesley J, et al. Identification of six new susceptibility loci for invasive epithelial ovarian cancer. Nat Genet. 2015;47(2):164-71.

37. Childs EJ, Mocci E, Campa D, Bracci PM, Gallinger S, Goggins M, et al. Common variation at 2p13.3, 3q29, 7p13 and 17q25.1 associated with susceptibility to pancreatic cancer. Nat Genet. 2015;47(8):911-6.

38. Wolpin BM, Rizzato C, Kraft P, Kooperberg C, Petersen GM, Wang Z, et al. Genome-wide association study identifies multiple susceptibility loci for pancreatic cancer. Nat Genet. 2014;46(9):994-1000.

39. Petersen GM, Amundadottir L, Fuchs CS, Kraft P, Stolzenberg-Solomon RZ, Jacobs KB, et al. A genome-wide association study identifies pancreatic cancer susceptibility loci on chromosomes 13q22.1, 1q32.1 and 5p15.33. Nat Genet. 2010;42(3):224-8.

40. Al Olama AA, Kote-Jarai Z, Berndt SI, Conti DV, Schumacher F, Han Y, et al. A meta-analysis of 87,040 individuals identifies 23 new susceptibility loci for prostate cancer. Nat Genet. 2014;46(10):1103-9.

41. Schumacher FR, Al Olama AA, Berndt SI, Benlloch S, Ahmed M, Saunders EJ, et al. Association analyses of more than 140,000 men identify 63 new prostate cancer susceptibility loci. Nat Genet. 2018.

42. Kote-Jarai Z, Olama AA, Giles GG, Severi G, Schleutker J, Weischer M, et al. Seven prostate cancer susceptibility loci identified by a multi-stage genome-wide association study. Nat Genet. 2011;43(8):785-91.

43. Eeles RA, Olama AA, Benlloch S, Saunders EJ, Leongamornlert DA, Tymrakiewicz M, et al. Identification of 23 new prostate cancer susceptibility loci using the iCOGS custom genotyping array. Nat Genet. 2013;45(4):385-91, 91e1-2.

44. Al Olama AA, Kote-Jarai Z, Giles GG, Guy M, Morrison J, Severi G, et al. Multiple loci on 8q24 associated with prostate cancer susceptibility. Nat Genet. 2009;41(10):1058-60.

45. Gudmundsson J, Sulem P, Gudbjartsson DF, Blondal T, Gylfason A, Agnarsson BA, et al. Genome-wide association and replication studies identify four variants associated with prostate cancer susceptibility. Nat Genet. 2009;41(10):1122-6.

46. Hoffmann TJ, Van Den Eeden SK, Sakoda LC, Jorgenson E, Habel LA, Graff RE, et al. A large multiethnic genome-wide association study of prostate cancer identifies novel risk variants and substantial ethnic differences. Cancer Discov. 2015;5(8):878-91.

47. Yeager M, Orr N, Hayes RB, Jacobs KB, Kraft P, Wacholder S, et al. Genome-wide association study of prostate cancer identifies a second risk locus at 8q24. Nat Genet. 2007;39(5):645-9.

48. Scelo G, Purdue MP, Brown KM, Johansson M, Wang Z, Eckel-Passow JE, et al. Genome-wide association study identifies multiple risk loci for renal cell carcinoma. Nat Commun. 2017;8:15724.

49. Henrion MY, Purdue MP, Scelo G, Broderick P, Frampton M, Ritchie A, et al. Common variation at 1q24.1 (ALDH9A1) is a potential risk factor for renal cancer. PLoS One. 2015;10(3):e0122589.

50. Purdue MP, Johansson M, Zelenika D, Toro JR, Scelo G, Moore LE, et al. Genome-wide association study of renal cell carcinoma identifies two susceptibility loci on 2p21 and 11q13.3. Nat Genet. 2011;43(1):60-5.

51. Henrion M, Frampton M, Scelo G, Purdue M, Ye Y, Broderick P, et al. Common variation at 2q22.3 (ZEB2) influences the risk of renal cancer. Hum Mol Genet. 2013;22(4):825-31.

52. Wu X, Scelo G, Purdue MP, Rothman N, Johansson M, Ye Y, et al. A genome-wide association study identifies a novel susceptibility locus for renal cell carcinoma on 12p11.23. Hum Mol Genet. 2012;21(2):456-62.

53. Ransohoff KJ, Wu W, Cho HG, Chahal HC, Lin Y, Dai HJ, et al. Two-stage genome-wide association study identifies a novel susceptibility locus associated with melanoma. Oncotarget. 2017;8(11):17586-92.

54. Barrett JH, Iles MM, Harland M, Taylor JC, Aitken JF, Andresen PA, et al. Genome-wide association study identifies three new melanoma susceptibility loci. Nat Genet. 2011;43(11):1108-13.

55. Falchi M, Bataille V, Hayward NK, Duffy DL, Bishop JA, Pastinen T, et al. Genome-wide association study identifies variants at 9p21 and 22q13 associated with development of cutaneous nevi. Nat Genet. 2009;41(8):915-9.

56. Bishop DT, Demenais F, Iles MM, Harland M, Taylor JC, Corda E, et al. Genome-wide association study identifies three loci associated with melanoma risk. Nat Genet. 2009;41(8):920-5.

57. Nan H, Xu M, Kraft P, Qureshi AA, Chen C, Guo Q, et al. Genome-wide association study identifies novel alleles associated with risk of cutaneous basal cell carcinoma and squamous cell carcinoma. Hum Mol Genet. 2011;20(18):3718-24.

58. Brown KM, Macgregor S, Montgomery GW, Craig DW, Zhao ZZ, Iyadurai K, et al. Common sequence variants on 20q11.22 confer melanoma susceptibility. Nat Genet. 2008;40(7):838-40.

59. Gudmundsson J, Thorleifsson G, Sigurdsson JK, Stefansdottir L, Jonasson JG, Gudjonsson SA, et al. A genome-wide association study yields five novel thyroid cancer risk loci. Nat Commun. 2017;8:14517.

60. Gudmundsson J, Sulem P, Gudbjartsson DF, Jonasson JG, Masson G, He H, et al. Discovery of common variants associated with low TSH levels and thyroid cancer risk. Nat Genet. 2012;44(3):319-22.

61. Gudmundsson J, Sulem P, Gudbjartsson DF, Jonasson JG, Sigurdsson A, Bergthorsson JT, et al. Common variants on 9q22.33 and 14q13.3 predispose to thyroid cancer in European populations. Nat Genet. 2009;41(4):460-4.

62. Figlioli G, Kohler A, Chen B, Elisei R, Romei C, Cipollini M, et al. Novel genome-wide association study-based candidate loci for differentiated thyroid cancer risk. J Clin Endocrinol Metab. 2014;99(10):E2084-92.
